# Utilization of proliferable extracellular amastigotes for transient gene expression, drug sensitivity assay, and CRISPR/Cas9-mediated gene knockout in *Trypanosoma cruzi*

**DOI:** 10.1371/journal.pntd.0007088

**Published:** 2019-01-14

**Authors:** Yuko Takagi, Yukie Akutsu, Motomichi Doi, Koji Furukawa

**Affiliations:** Biomedical Research Institute, National Institute of Advanced Industrial Science and Technology, Tsukuba, Ibaraki, Japan; New York University School of Medicine, UNITED STATES

## Abstract

*Trypanosoma cruzi* has three distinct life cycle stages; epimastigote, trypomastigote, and amastigote. Amastigote is the replication stage in host mammalian cells, hence this stage of parasite has clinical significance in drug development research. Presence of extracellular amastigotes (EA) and their infection capability have been known for some decades. Here, we demonstrate that EA can be utilized as an axenic culture to aid in stage-specific study of *T*. *cruzi*. Amastigote-like property of axenic amastigote can be sustained in LIT medium at 37°C at least for 1 week, judging from their morphology, amastigote-specific UTR-regulated GFP expression, and stage-specific expression of selected endogenous genes. Inhibitory effect of benznidazole and nifurtimox on axenic amastigotes was comparable to that on intracellular amastigotes. Exogenous nucleic acids can be transfected into EA via conventional electroporation, and selective marker could be utilized for enrichment of transfectants. We also demonstrate that CRISPR/Cas9-mediated gene knockout can be performed in EA. Essentiality of the target gene can be evaluated by the growth capability of the knockout EA, either by continuation of axenic culturing or by host infection and following replication as intracellular amastigotes. By taking advantage of the accessibility and sturdiness of EA, we can potentially expand our experimental freedom in studying amastigote stage of *T*. *cruzi*.

## Author summary

We developed an experimental system to study amastigote stage of *Trypanosoma cruzi* as a proliferable axenic culture. Use of axenic amastigotes allows us to directly introduce exogenous gene into *T*. *cruzi* amastigote and select for drug resistant parasite to enrich the transfectants. Our strategy bypasses differentiation steps involved in conventional epimastigote transfection procedure to obtain transgenic amastigotes. Gene knockout can also be performed in amastigote-specific manner, using Cas9-expressing extracellular amastigotes. Drug sensitivity could also be assessed during 1-week axenic growth period. Our method potentially leads to variety of new experimental strategies to make amastigote-stage-specific manipulations and analyses possible.

## Introduction

*Trypanosoma cruzi* is the causative agent of Chagas’ disease, which affects 6–7 million people mainly in Latin America[1]. The parasite is transmitted by a reduviid bug through its contaminated feces, and enters into the mammalian host when the bite site is rubbed or scratched. Chagas’ disease can also be acquired by vertical and perinatal transmissions, blood transfusion or organ transplant, and oral transmission through contaminated food[2,3]. Approximately 30 to 40% of infected people develop chronic disease 10 to 30 years after acute infection[1]. Common chronic phase manifestations include cardiomyopathy, arrhythmias, megaviscera, and polyneuropathy.

There are currently two drugs available to treat Chagas’ disease; benznidazole and nifurtimox. Both drugs are effective in acute phase of the infection, but efficacy becomes limited once the disease proceeds to chronic phase[4]. Because most parasite carriers do not get a timely diagnosis or have access to the medication, many of them proceed to chronic phase unnoticed or without proper treatment. In addition, aforementioned drugs are known to cause adverse side effects in roughly 40% of the patients[1]. Thus, development of safer new drugs that are effective in chronic phase is a pressing matter.

*T*. *cruzi* has distinct developmental stages in its life cycle (Reviewed in [5]). In a reduviid bug, the parasite replicates in a form called epimastigote. It differentiates into metacyclic trypomastigote, a non-replicative infectious form, in the insect rectum before being defecated. Trypomastigotes enter a mammalian host and invade the host cell, where they transform into flagella-less amastigotes and replicate intracellularly. Amastigotes differentiate into highly motile blood stream trypomastigotes as they emerge out of the host cells. The parasites then travel through blood stream to infect another host cell, or to be picked up by an insect vector to complete the life cycle.

In order to develop a chemotherapeutic agent effective in chronic phase of the disease, it is important to identify a drug target that is essential for the parasite in amastigote stage. Yet, it is difficult to perform a knockout study in this stage of *T*. *cruzi* due to inaccessibility of the parasite in the host cells. Accordingly, conventional method to obtain a transgenic amastigote starts with epimastigote transfection. However, this procedure potentially introduces unwanted bias in the resulting amastigote population by selecting for transfectants that are better-fitted in epimastigote or trypomastigote stages. Even though transient transfection of trypomastigote allows to bypass metacyclogenesis step[6], it still requires active invasion of the host cells and trypomastigote-amastigote differentiation, which still may introduce some bias.

To overcome this issue, we focused our attention on extracellular amastigote (EA) to utilize it as a tool for direct experimental manipulations. EA can be obtained either by spontaneous appearance in *T*. *cruzi*-host co-culture, or by inducing differentiation of trypomastigote *in vitro* at low pH[7]. It has been reported that EA is morphologically very similar to intracellular amastigote[7–10] and expresses surface glycoprotein SSP-4, which is a hallmark of amastigote stage parasite[7,9,11]. EA is capable of infecting culture host cells by inducing the host actin polymerization[12,13], even if the host cell is not a professional phagocyte[8,10,12–15]. Notably, EA is able to establish infection and kill mice when inoculated intraperitoneally[8], and free amastigotes can be found in blood stream of infected animals[9]. Traditionally, amastigotes were considered to be non-infectious, but above findings shed light on the important role of EA in *T*. *cruzi* dissemination process. Molecular mechanisms of EA take-up by the host cells are beginning to be elucidated as well[16–20].

In the present study, we demonstrate that EA can replicate free of host cells and can be utilized in variety of assays, including exogenous gene expression and CRISPR/Cas9-mediated gene knockout. We also show that susceptibility of proliferating axenic amastigotes to benznidazole and nifurtimox is close to that of intracellular amastigotes in host 3T3 cells. Our strategy to utilize EA expands methodological freedom in amastigote study, and contributes to advance our understanding in this pathogenic stage of *T*. *cruzi*.

## Methods

### *T*. *cruzi* culture

Epimastigote of *T*. *cruzi* Tulahuen strain (provided by Dr. Takeshi Nara, Juntendo University) was maintained in liver infusion tryptose (LIT) medium supplemented with 10% heat-inactivated FBS at 28°C. Metacyclogenesis was performed in RPMI medium [21], and differentiated metacyclic trypomastigotes were isolated by DEAE ion-exchange chromatography[22]. Metacyclic trypomastigotes were added to the 3T3-Swiss Albino fibroblast cell culture for infection, and amastigote-containing culture was maintained in DMEM supplemented with 10% FBS and penicillin/streptomycin at 37°C under 5% CO_2_ in a humidified incubator, until tissue-derived trypomastigotes emerged out to the culture supernatant.

Axenic amastigotes were obtained by *in vitro* amastigogenesis according to the method described by Tomlinson *et al*.[7] The tissue-derived trypomastigotes were collected from culture supernatant by centrifugation at 2000 ×g for 15 min, and were transformed into amastigotes by incubation in DMEM buffered with 20 mM MES (pH 5.0) and supplemented with 0.4% bovine serum albumin (BSA) for 24 h at 37°C.

To obtain intracellularly-derived amastigotes, infected 3T3 cells were detached from a culture flask by trypsin treatment, and centrifuged at 100 ×g for 5 min. The cells were washed by PBS to remove trypomastigotes and EA, and were suspended in 1 mL of Phosphate Saline Glucose buffer (1:9 mixture with 1% glucose)[23]. To release intracellular amastigotes, the cell suspension was passed through a syringe with 27 G needle 40 times to lyse the host 3T3 cells. Unbroken host cells and debris were removed by centrifugation at 100 ×g for 3 min. The intracellular amastigotes in the supernatant were purified by anion-exchange chromatography[23].

### Microscopy

All-in-One Fluorescence Microscope BZ-X710 (Keyence Co. Ltd., Japan) was used to capture images of parasites, using appropriate filter sets. For objective lens, S PL FL ELWD ADM 20xC (NA0.45) and CFI Plan Apo λ60xH (NA1.40) Nikon lenses were used.

### RT-qPCR of stage-specific mRNAs

TRIzol Reagent (Thermo Fisher Scientific Inc., USA) was used to extract total RNA from epimastigote, tissue-derived trypomastigote, intracellular amastigote, EA derived by *in vitro* amastigogenesis, and axenic amastigotes cultured in LIT medium for 1, 3, 5, and 7 days. Extracted RNAs were treated with DNase I (Thermo Fisher Scientific) to eliminate potential genomic DNA contamination, and the samples were purified by phenol/chloroform/isoamyl alcohol extraction and ethanol precipitation. Reverse-transcription was performed by using 1 μg of above RNAs, 0.5 μg of Oligo(dT)_12-18_ Primer (Thermo Fisher Scientific), and Superscript III (Thermo Fisher Scientific). cDNA was synthesized at 50°C for 1 h, and the enzyme was heat-inactivated at 70°C for 15 min. The reaction product was diluted 10 fold before being used for the following qPCR assay.

Target gene IDs and sequences of primers used for qPCR are listed in S1 Table. For amplification reaction, TB Green Premix Ex Taq II (Tli RNaseH Plus) (Takara Bio Inc., Japan) was mixed with 2 μL of diluted cDNA, 0.2 μM each gene-specific forward and reverse primers, and ROX reference dye in the total reaction volume of 20 μL. qPCR was performed according to the manufacturer’s instruction by using StepOnePlus Real-Time PCR System (Thermo Fisher Scientific). Specificity of the reaction was verified by melting curve analysis, and the gene expression was quantitated by relative standard curve method using StepOne Software v2.3. Expression levels were normalized by GAPDH as an internal control.

### Drug sensitivity assay with axenic amastigote

Benznidazole (Sigma-Aldrich Co. LLC, USA) and nifurtimox (Sigma-Aldrich) were dissolved in DMSO and dispensed into 96 well microplate, and *T*. *cruzi* axenic amastigotes in LIT medium (1 × 10^6^ cells in 100 μL) was added to each well. The final concentration of DMSO was 0.5%. After 48 h of incubation at 37°C under 5% CO_2_ in a humidified incubator, 10 μL of resazurin solution (Sigma-Aldrich) was added at a final concentration of 3 mM[24]. The plates were incubated for additional 5 h, and the reaction was stopped by addition of 50 μL 3% SDS. Amount of resorufin was quantitated by scanning the microplate by SpectraMax Gemini fluorescent plate reader (Molecular Devices, LLC., USA) at ex. 560 nm/em. 590 nm. EC50 was calculated by fitting the dose response curves with non-linear regression analysis, using "(inhibitor) vs. normalized response" model of GraphPad Prism7 software (GraphPad Software Inc., USA).

### Drug sensitivity assay with intracellular amastigote

Host 3T3 cells were seeded onto 96-well black, clear bottom microplate (Corning Inc., USA) at 5 × 10^3^ cells/well in 100 μL of DMEM supplemented with 10% FBS for 4 h to allow cell attachment. Tissue-derived trypomastigotes were added at multiplicity of infection (MOI) of 20, and incubated at 37°C for 24 h. Uninfected trypomastigotes were removed by washing with PBS, followed by addition of 100 μL of DMEM (10% FBS) containing benznidazole or nifurtimox dissolved in DMSO. The final concentration of DMSO was 0.5%. After 48 h incubation at 37°C, the media was removed from wells and cells were fixed by addition of 100 μL of 4% formaldehyde for 15 min at room temperature. After fixation, the nuclei were stained by adding 100 μL of 1.0 μg/mL Hoechst 33342 (Thermo Fisher Scientific) and 0.05% Triton X-100 (Wako Pure Chemical Industries, Ltd., Japan) for 15 min, followed by washing wells with PBS 4 times. The plates were imaged by a fluorescence microscopy. Host cells containing more than three amastigotes were considered as infected. EC50 was calculated by fitting the dose response curves with non-linear regression analysis, using “(inhibitor) vs. normalized response" model of GraphPad Prism7 software.

### Plasmid construction

The plasmid constructs and the corresponding cell lines are summarized in Supplementary S1 Fig. Expression vector pTREX-attR derived from pTREX-n[25] was provided by Dr. Takeshi Nara. For constitutive expression of EGFP, EGFP gene was amplified by PCR using forward (5’-CTCTAGAATGGTGAGCAAGGGCGAGGAGCT-3’) and reverse (5’- GCTCGAGTTACTTGTACAGCTCGTCCATGCC-3’) primers, and ligated into XbaI and XhoI sites of pTREX-attR to generate pTREX-EGFP. For construction of amastigote-specific EGFP expression vector pTREX-EGFP-amastin 3’UTR, the fragment containing amastin 3’UTR upstream of tuzin site[26] (GenBank: U25030.1) was amplified using forward (5’-GTACAAGTAACTCGAGCGGGTGCATCCACCGTCT-3’) and reverse (5’-TCGTAAATGGCTCGAGCGCAGGGCGGGCAGCGGC-3’) primers. The resulting 800 bp fragment was ligated into 3’ end of the EGFP gene at XhoI site using In-Fusion HD cloning kit (Takara Bio). *Streptococcus pyogenes* Cas9 sequence (RefSeq. NC_002737), twice-repeated sequence of the SD40 nuclear localization signals and EGFP sequence were synthesized and ligated into XbaI and XhoI site of pTREX-attR to generate pTREX-Cas9-EGFP. Amastin 3’UTR sequence was inserted into pTREX-Cas9-EGFP as described above to generate pTREX-Cas9-EGFP-amastin-3’UTR. To generate pTREX-mDsRed-Bsd plasmid for expression of mDsRed and blasticidin resistant gene, full-length mDsRed was amplified by PCR using forward (5’-TGCTCTAGAATGGCCTCCTCCGAGAACGT) and reverse (5’-CCGCTCGAGCTACAGGAACAGGTGGTGGC) primers, and resulting fragment was subcloned between XbaI and XhoI sites. Neomycin resistance gene was then replaced by blasticidin selection marker[27] at PspXI and NheI sites.

### Plasmid transfection

Transfection was carried out using Basic Parasite Nucleofector Kit 2 (Lonza Inc. Switzerland). Briefly, 2 × 10^7^ epimastigote cells in their log phase were suspended in 100 μL of Nucleofector buffer with provided supplement solution, and 20 μg of plasmid was added to the mixture. Electroporation was carried out using program X-14 of Amaxa Nucleofector device (Lonza) unless otherwise stated. To generate a stable cell line harboring pTREX-EGFP-amastin-3’UTR (EGFP-ama), pTREX-Cas9-EGFP (Cas9) and pTREX-Cas9-EGFP-amastin-3’UTR (Cas9-ama), the transfectants were selected in LIT medium containing 500 μg/mL G418 for over 4 weeks at 28°C.

For transient expression of mDsRed, axenic amastigotes were electroporated with pTREX-mDsRed-Bsd plasmid as described above. The transfected amastigotes were cultured in LIT medium at 37°C under 5% CO_2_ in a humidified incubator, or applied onto 3T3 cell culture for an infection experiment. To enrich positive transfectants by blasticidin S resistant marker, axenic amastigotes were transferred to LIT medium immediately after electroporation, and 50 μg/mL of blasticidin S (Wako Pure Chemical Industries) was added 24 h later. Cells were monitored for the next 6 days for mDsRed expression, and fraction of mDsRed positive amastigotes were quantitated under fluorescence microscopy. Blasticidin-selected transgenic amastigotes were subsequently applied onto 1 × 10^5^ cells of host 3T3 in 24-well plate at MOI of 40. After 2 days of incubation, the cells were washed 3 times with DMEM to remove amastigotes remained outside of the host cells. Replication of internalized mDsRed-positive amastigotes was monitored for the next 2 days under fluorescence microscopy.

### CRISPR/Cas9-mediated gene knockout

gRNA was purchased from IDT (Integrated DNA Technologies, Inc., USA) as two synthetic RNA oligonucleotides, Alt-R CRISPR crRNA and tracrRNA. The target sequences of EGFP, TcCgm1 and mDsRed (negative control) were GGTGGTGCAGATGAACTTCA, TAGCCGCGATGGAGAGTTTA and GGACGGCACCTTCATCTACA, respectively. Gene-specific crRNA and universal tracrRNA were annealed to make a complete gRNA, according to company’s protocol. Transfection of gRNA into Cas9-expressing epimastigote was carried out essentially in the same manner as plasmid transfection described above, except 5 μg gRNA was used. For amastigote transfection, 1 × 10^7^ Cas9-ama-expressing EAs were collected immediately after amastigogenesis in pH 5.0, and were resuspended in Nucleofector buffer. After the electroporation, EAs were transferred to 5 mL of LIT medium and incubated at 37°C under 5% CO_2_. Cell growth was monitored by counting surviving cell number using Burker-Turk hemocytometer. Propidium iodide was mixed to the EA culture prior to the counting to aid in distinguishing viable amastigotes from dead parasites.

For quantitation of EGFP knockout efficiency, percentage of EGFP-positive population was calculated by analyzing the total cell count in bright field images and the EGFP-positive cell count in fluorescent images, using Hybrid Cell Count software of Keyence microscope. Fluorescence intensity of EGFP-positive/negative cut-off was determined by analyzing the images of WT parasites as the background fluorescence. Total of 1000 parasites were analyzed for transfected epimastigotes, and 500 were analyzed for transfected amastigotes.

### Guanylyltransferase assay

gRNA-transfected Cas9 cells or Cas9-ama cells were harvested at 2 days after electroporation. Cell pellets were stored at -80°C until use. The cells were resuspended in a buffer containing 50 mM Tris-HCl (pH 7.5), 20 mM NaCl, 10% sucrose, 0.1% Triton X-100 and 1×cOmplete EDTA-free Protease Inhibitor Cocktail (F. Hoffmann-La Roche, Ltd., Switzherland) for lysis, and cell debris were cleared by a centrifugation for 1 min at 8000 rpm. Guanylyltransferase assay was carried out by incubating 4 μg of cleared lysate in a reaction mixture containing 50 mM Tris-HCl (pH 7.5), 10 mM MgCl_2_, 2 mM DTT and 40 μM [α-^32^P]-GTP for 20 min at 30°C[28]. The reaction was terminated by addition of SDS loading buffer, and the products were resolved on a 10% SDS-PAGE. Radiolabeled enzyme-GMP covalent adducts were visualized by BAS-2500 phosphorimager and quantitated by Image Gauge 4.0 software (Fujifilm Corp., Japan).

## Results

### Proliferation of *T*. *cruzi* amastigote as axenic culture

**Fig 1:**
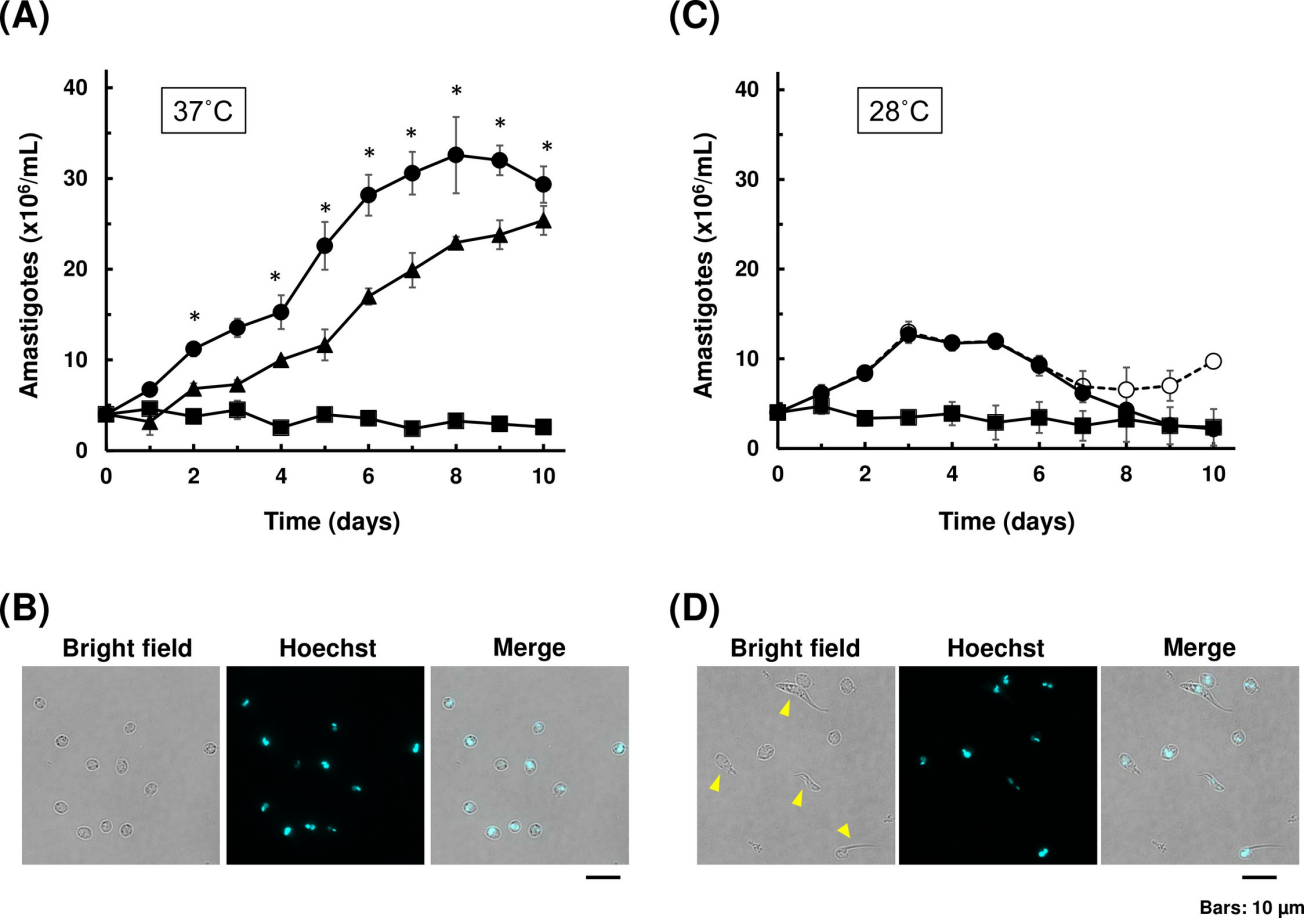
Growth and morphology of axenic amastigote culture. (A) Growth curves of axenic amastigotes at 37°C. Amastigotes derived from *in vitro* amastigogenesis were cultured in either LIT medium (closed circle) or DMEM (closed square) at 37°C. Intracellular amastigotes isolated from host 3T3 cells were also cultured in LIT medium (closed triangle). The number of amastigotes were counted under microscopy. At least two independent experiments were performed in triplicates for each group, and mean values (±SD) of one representative experiment were plotted. Significant difference compared to DMEM was indicated (*p<0.05, Kruskal-Wallis test). (B) Morphology of amastigote after 10 days of axenic cultivation in LIT medium at 37°C. The parasites were fixed and stained using Hoechst 33342 to detect nucleus and kinetoplast. Scale bar: 10 μm. (C) Growth curves of axenic amastigotes at 28°C. Amastigotes derived from *in vitro* amastigogenesis were cultured in either LIT medium (closed and open circles) or DMEM (closed square) at 28°C. For LIT 28°C, closed circle represents the number of round form amastigote only, and open circle represents the total number of parasites including intermediate morphologies. At least two independent experiments were performed in triplicates for each group, and mean values (±SD) of one representative experiment were plotted. No significant difference was observed among groups (Kruskal-Wallis test). (D) Representative image of parasites displaying intermediate morphology after 10 days of axenic cultivation at 28°C (open circle in (C)). Parasites with intermediate forms are indicated by arrow heads.

We first tested the multiplication capability of amastigote outside of the host cell. EA was obtained by differentiation of tissue-derived trypomastigotes by incubating the parasite in acidic DMEM, buffered with MES (pH 5.0) and supplemented with 0.4% BSA, for 24 h at 37°C. EA was subsequently cultured in LIT medium (10% FBS) or DMEM (10% FBS) at 28°C or 37°C, and their growth was monitored for the next 10 days. Axenic amastigotes replicated most efficiently in LIT medium at 37°C (Fig 1A, closed circle). In this condition, amastigotes continued to proliferate for a week before replication slowed down and eventually ceased past day 8. Intracellular amastigotes obtained by host cell rupture also replicated in LIT medium at 37°C, and showed similar growth pattern as *in vitro*-derived EA (Fig 1A, closed triangle). DMEM at 37°C did not support the growth of axenic amastigote (Fig 1A closed square). At this temperature, amastigotes in all groups retained typical round morphology throughout the observation period (Fig 1B). On the other hand, EAs replicated only for a few days in LIT medium when incubated at 28°C (Fig 1C, closed circle). In addition, some amastigotes started to transform into intermediate morphologies on day 5, which resembles epimastigote, trypomastigote and spheromastigote, based on their shapes and nuclear staining patterns (Fig 1D). By day 8, the number of those intermediate forms were up to 30% of the total number of the parasites in LIT 28°C (Fig 1C, closed and open circles). Axenic amastigotes in DMEM at 28°C did not replicate, although their morphology remained as round form throughout the observation period (Fig 1C, closed square). These results are consistent with previously reported observations that EA can replicate free of host cells, given appropriate media condition and temperature setting[10,29,30]. We also found that amastigotes derived from both *in vitro* amastigogenesis and from host cell rupture have comparable proliferation capability in axenic environment (Fig 1A, closed circle and triangle).

For the rest of experiments in this paper, we used LIT medium at 37°C to maintain axenic amastigote culture. We use the term “EA” for extracellular amastigote in general or amastigote soon after *in vitro* amastigogenesis, and “axenic amastigote” for amastigote cultured free of host cells for more than two days.

### Stage-specific gene expression is sustained in axenic amastigotes

**Fig 2:**
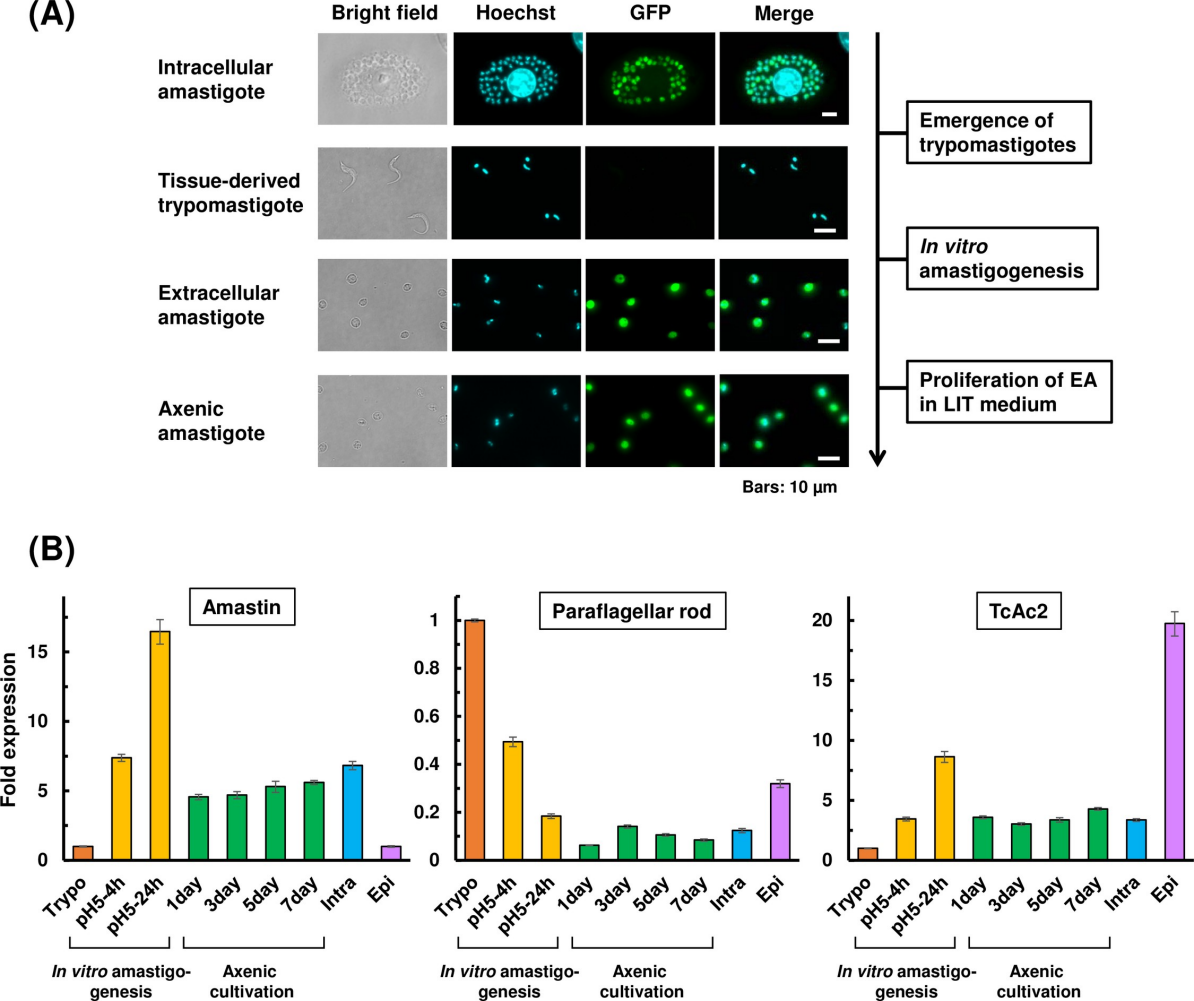
UTR-regulated GFP expression and differentially expressed endogenous genes in axenic amastigotes. (A) EGFP expression of EGFP-ama cell line in intracellular amastigote, tissue-derived trypomastigote, EA produced by *in vitro* amastigogenesis, and axenic amastigote after 5 days of LIT cultivation. Scale bars: 10 μm. (B) Expression levels of stage-specific genes analyzed by RT-qPCR. Total RNA was extracted from trypomastigote (Trypo), transforming trypomastigote in DMEM (pH 5.0) during *in vitro* amastigogenesis at 4 h (pH5-4h) and at 24 h (pH5-24h), axenic amastigote maintained in LIT medium at 37°C for indicated time period (1day, 3day, 5day, and 7day), intracellular amastigote harvested by host cell lysis (Intra), and epimastigote (Epi). Target genes, δ-amastin, paraflagellar rod protein and TcAc2, were previously shown to be upregulated in amastigote, trypomastigote and epimastigote stages, respectively[33,34]. The fold expression is normalized to Trypo. Two independent experiments were performed to ensure the reproducibility, and values from one representative experiment are plotted. The error bar represents SD of qPCR reactions performed in triplicates.

Since trypanosomatids transcribe their mRNAs polycistronically, the amount of each mRNA is controlled mainly post-transcriptionally, and 3’UTR plays a major role in differential gene expression in trypanosomatids (Reviewed in [31]). Amastin is a family of surface glycoproteins, which is most abundantly expressed in amastigote stage of *T*. *cruzi*[32]. To investigate whether axenic amastigotes maintain amastigote-specific 3’UTR-mediated gene regulation during prolonged cultivation, we generated a cell line that expresses EGFP under the control of amastin 3’UTR[26]. Control cells harboring EGFP construct without stage-specific 3’UTR expressed EGFP in all developmental stages (S2 Fig, EGFP), whereas EGFP-amastin 3’UTR cell line (EGFP-ama) expressed EGFP in intracellular amastigote stage but not in epimastigote or trypomastigote stages (S2 Fig, EGFP-ama). When non-fluorescent, tissue-derived trypomastigotes of EGFP-ama cell line were transformed into EA by *in vitro* amastigogenesis, EGFP signal became apparent as trypomastigotes differentiated into round flagella-less form (Fig 2A, Extracellular amastigote). Proliferating axenic amastigotes in LIT medium continued to express EGFP, and retained the fluorescence at the same level even after 1-week of host-free replication (Fig 2A, Axenic amastigote). These results indicate that *in vitro*-transformed EA and axenic amastigotes use similar differential gene expression system to that of intracellular amastigote, and that amastigote-specific 3’UTR regulation persists even after 1 week of axenic cultivation.

To verify whether the endogenous genes in axenic amastigote are also under stage-specific regulation, the amount of selected mRNAs; amastin, paraflagellar rod protein and TcAc2, were analyzed by RT-qPCR. Differential expression of the target genes were microarray-identified and qPCR-verified previously[33] and analyzed by RNA-seq more recently[34] by other groups. δ-Amastin is known to be expressed abundantly in amastigote stage[35]. In axenic amastigote, mRNA of δ-amastin remained in similar level to that in intracellular amastigote isolated from host cells throughout 1 week of axenic cultivation, which is around 4–6 fold higher than the level of trypomastigote or epimastigote (Fig 2B). Remarkable upregulation of δ-amastin mRNA during *in vitro* amastigogenesis is consistent with RNA-seq data, in which peak expression of δ-amastin coincides with the timing of trypomastigote-amastigote transition[34], and the fact that the surface of EA is already rich in amastigote-specific glycoproteins by the time it finishes differentiation from trypomastigote[7,9]. Paraflagellar rod protein, a key component of flagellum, was significantly downregulated as trypomastigote transformed into EA, and remained roughly 10 fold less than that of trypomastigote during axenic cultivation (Fig 2B). This low expression in axenic amastigote was roughly the same level in intracellular amastigote, reflecting their flagellar-less morphology. TcAc2 is a thiol-dependent reductase and is a virulence factor, also known as Tc52[36]. It was significantly upregulated in epimastigote, comparing to trypomastigote, axenic amastigote and intracellular amastigote, as expected from the previous report[33,34]. We observed temporal upregulation of TcAc2 during *in vitro* amastigogenesis, which is presumably due to a stress response of the parasite to acidic environment[36]. In all three target genes examined, expression levels in axenic amastigote was comparable to that in intracellular amastigote, and clearly distinct from trypomastigote and epimastigote, even after 1 week of host-free replication.

### Effect of trypanocidal compounds on axenic amastigotes

To further characterize the nature of proliferating axenic amastigotes and to explore its potential usage in drug screening assay, we compared the efficacy of benznidazole and nifurtimox, clinical drugs for Chagas’ disease, against axenic amastigotes and intracellular amastigotes. We employed resazurin redox assay to quantitate the cell viability of axenic culture. For intracellular amastigote assay, host-amastigote co-cultures were fixed and stained by Hoechst to identify infected 3T3 cells. Percent infection was calculated and were normalized to untreated controls to derive the relative inhibition.

**Fig 3:**
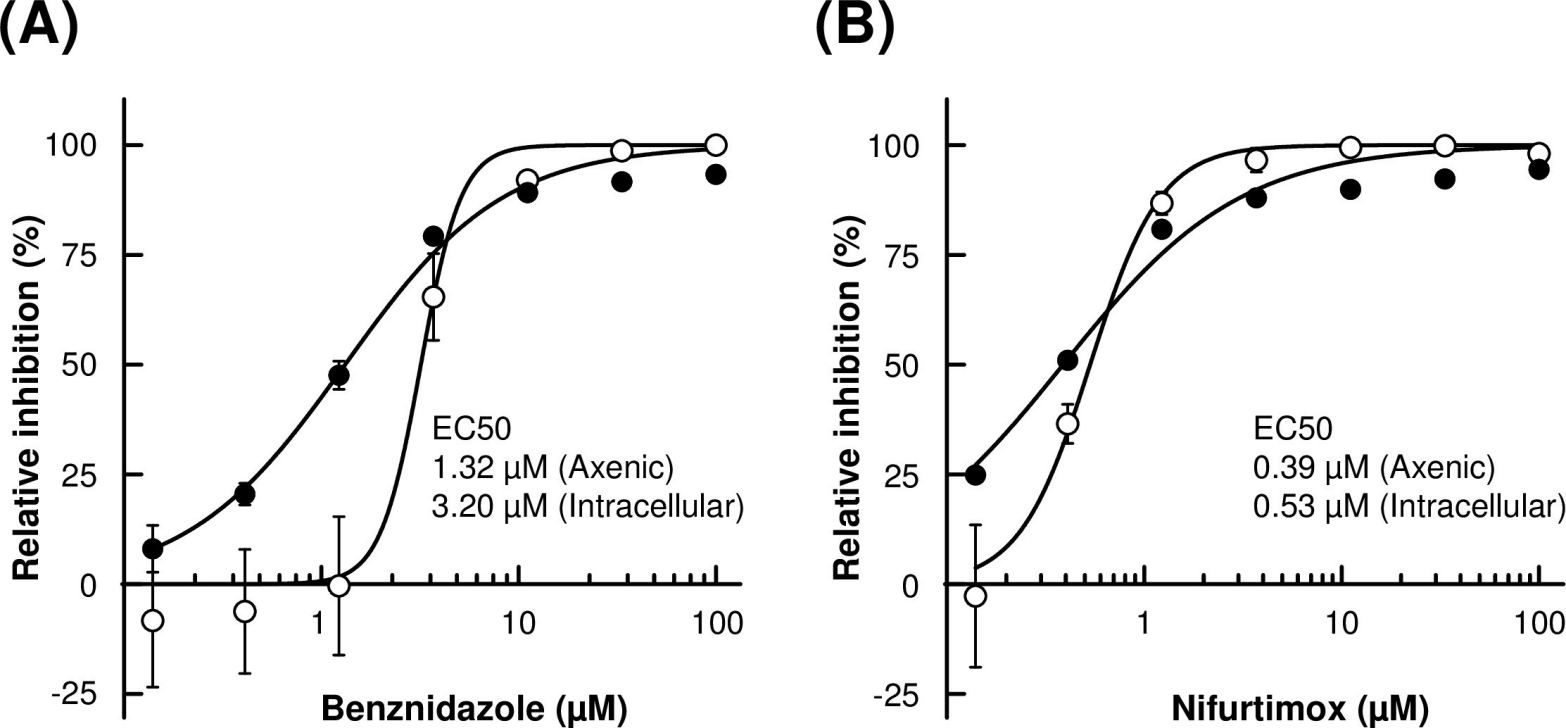
Effect of benznidazole and nifurtimox on axenic amastigotes and 3T3 intracellular amastigotes. Indicated concentration of benznidazole (A) or nifurtimox (B) was added to the culture of axenic amastigotes (closed circle) or 3T3 intracellular amastigotes (open circle). Growth of axenic culture was quantitated by resazurin assay. Relative inhibition was calculated from Relative Fluorescent Units and normalized to no drug control. Growth of intracellular amastigotes in the host 3T3 cells was calculated from the ratio of infected over uninfected host 3T3 cells and normalized to no drug control. Experiment was performed in triplicate, and mean values (±SD) were plotted.

Intracellular amastigote assay (Fig 3, open circle) provided EC50s of 3.20 (± 0.36) μM and 0.53 (± 0.031) μM for benznidazole and nifurtimox, respectively, which are comparable to previously reported EC50s[37]. Estimated EC50s of benznidazole and nifurtimox for axenic amastigote (Fig 3, closed circle) were 1.32 (± 0.074) μM and 0.39 (± 0.028) μM, respectively. Dose response curves of intracellular amastigotes tend to show steeper Hill Slope than axenic amastigotes, both for benznidazole and nifurtimox. Nonetheless, these results indicate that axenic amastigotes and intracellular amastigotes have similar susceptibility to the tested trypanocidal compounds.

### Exogenous gene expression in EA via conventional electroporation

**Fig 4:**
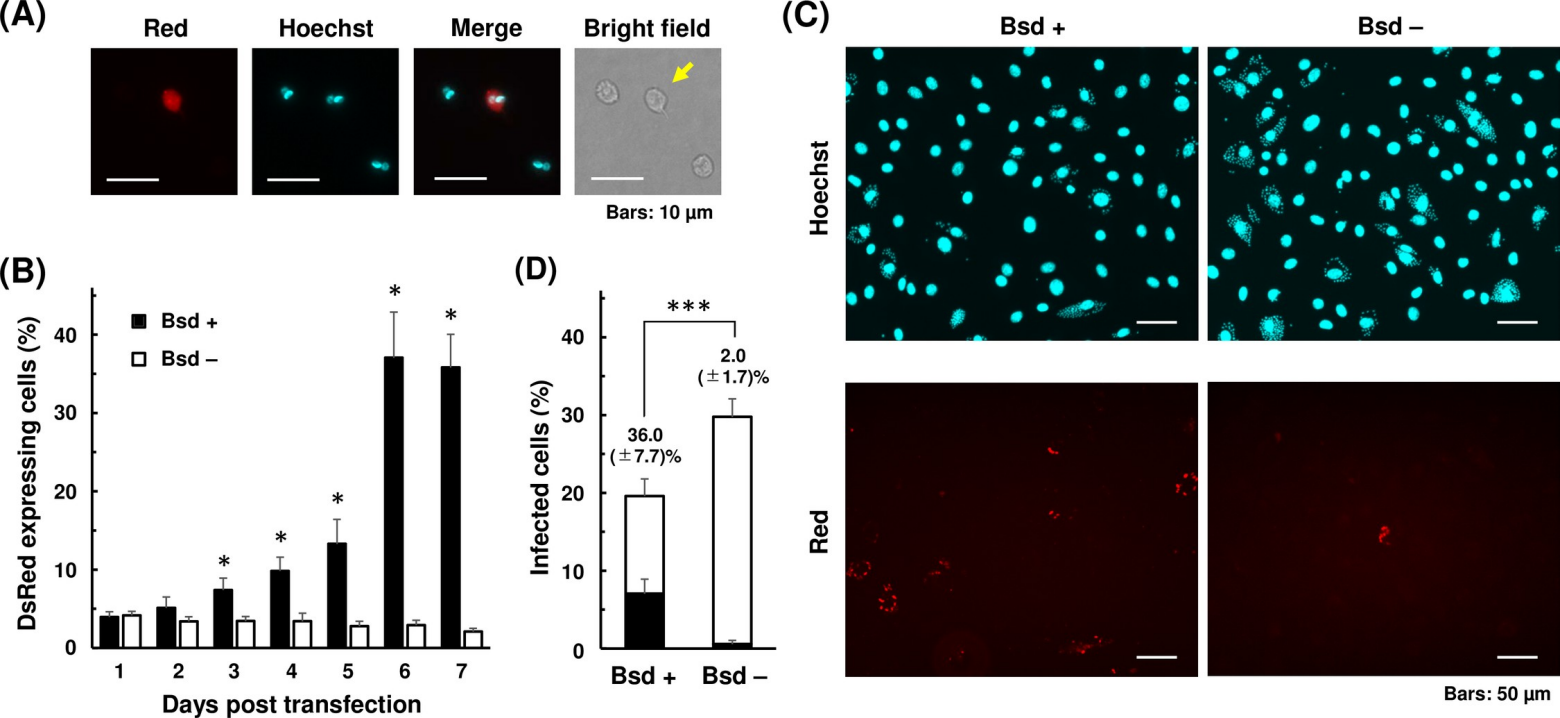
Expression of mDsRed and blasticidin selection in axenic amastigotes. (A) Axenic amastigotes were transfected with 20 μg of pTREX-mDsRed-Bsd plasmid using X-14 program of Amaxa Nucleofector, and maintained in LIT (10% FBS) at 37°C. Expression of mDsRed was visualized under fluorescence microscope 1 day after electroporation. An arrow indicates transgenic amastigote. Scale bars: 10 μm. (B) Blasticidin S was added 1 day after electroporation to select for the positive transfectants. Number of amastigotes was counted every day for the next 6 days. Electroporation and the following selection were performed in triplicates. Mean percentages (±SD) of mDsRed-expressing amastigotes in blasticidin added (Bsd +) and not added (Bsd -) cultures are plotted (*p<0.05, Mann-Whitney *U* test). Three independent experiments were performed to ensure reproducibility, and the result of one representative experiment is shown. (C) Cultivated axenic amastigotes in LIT medium with (+) and without (–) blasticidin on day 6 in (B) were used to infect 3T3 cells. Representative images from 4 days post infection are shown. Scale bars: 50 μm. (D) Percentage of infected 3T3 cells containing mDsRed-expressing (black bar) and not expressing (white bar) amastigotes. Host cell containing ≥3 amastigotes was defined as infected. Values represent the average (±SD) of at least six countings from duplicated experiments, using microscopy images captured at different locations of culture wells (***p<0.001, Mann-Whitney *U* test).

Taking advantage of the fact that axenic amastigote is fairly robust, we explored the possibility of using EAs for exogenous DNA transfection by standard electroporation method. Nucleofector system was used as electroporation device and reagent, and pTREX-mDsRed-Bsd plasmid was used to visualize transient expression of a fluorescent marker, mDsRed. Expression of mDsRed became visible 1 day after electroporation (Fig 4A), and continued to be detectable at least for the next 6 days. Eight pre-programed pulse settings (U-06, U-33, V-06, V-33, X-01, X-06, X-14, and Y-01) were tested to determine suitable pulse condition for EA electroporation. Highest transfection efficiency, 7.4 ±0.8%, was achieved by X-14 program, whereas maximum survival rate was observed with X-01 and X-06 programs (S2 Table). We selected X-14 program as our standard protocol for EA transfection, as it is also suited for epimastigote transfection[38].

Since axenic amastigote culture is sustainable at 37°C for approximately 1 week without major deterioration (Fig 1), we next subjected the mDsRed-Bsd transfected EAs to blasticidin selection to enrich mDsRed-positive population. After pTREX-mDsRed-Bsd plasmid was electroporated into EAs, the parasites were transferred to LIT medium and cultured at 37°C. Blasticidin S was added 24 h later, and the percentage of mDsRed-expressing amastigotes were monitored for the next 6 days. Without addition of blasticidin, fraction of mDsRed-positive amastigotes gradually decreased after transfection (Fig 4B, Bsd -). On the other hand, percentage of mDsRed-positive amastigotes increased in presence of blasticidin, since many of mDsRed-negative amastigotes died during the selection period (Fig 4B, Bsd +). Effect of drug selection peaked on 5 days after blasticidin addition, or 6 days post electroporation. Further selection beyond 5 days did not seem to benefit the population enrichment. This is primarily due to gradual loss of proliferation capability of axenic amastigote after 1 week of cultivation, as seen in Fig 1A.

Next, blasticidin-selected transfectants from above were used to infect 3T3 cells. Axenic amastigotes were allowed to infect host cells for two days, and amastigotes remained outside of the host cells were washed away. The host-parasite co-culture was incubated for additional two days before visualizing and quantitating the prevalence of mDsRed-expressing amastigotes in 3T3 cells. Transfectants were successfully internalized by the host cells and established productive infection, despite the 6-day-long axenic cultivation (Fig 4C). Infection efficiencies of blasticidin-selected (Bsd +) and non-selected (Bsd -) transfectants were 19.6% and 29.8%, respectively (Fig 4D). When blasticidin-selected EAs were used for infection, percentage of mDsRed-expressing amastigotes in total intracellular amastigotes was 36.0%, whereas that of non-selected amastigotes was only 2.0% (Fig 4D, black bars). These proportions are well correlated with the fractions of mDsRed-positive population in initial transfectants applied onto 3T3 cells for infection (Fig 4B).

Blasticidin-selected amastigotes differentiated into trypomastigotes and emerged out to culture supernatant 4 days post infection (S1 and S2 Movies). These results suggest that axenic amastigotes can be utilized for electroporation-mediated exogenous gene transfer, and selectable marker is useful to enrich positive transfectants without significantly impairing the ability of amastigotes to infect host cells and to proceed to the next stage of life cycle.

### CRISPR/Cas9-mediated gene knockout in EA

Drug target research against *T*. *cruzi* entails validation of gene essentiality especially in amastigote stage. Even though CRISPR/Cas9 system offers effective knockout strategy in *T*. *cruzi*[27,39–44], it is troublesome to perform this in amastigotes, because intracellular amastigotes are shielded by the host cell and direct access for experimental manipulation is hindered. Utilization of EA as an experimental tool potentially offers an alternative mean to bypass such issue, and allows us to perform knockout studies solely in amastigote stage.

**Fig 5:**
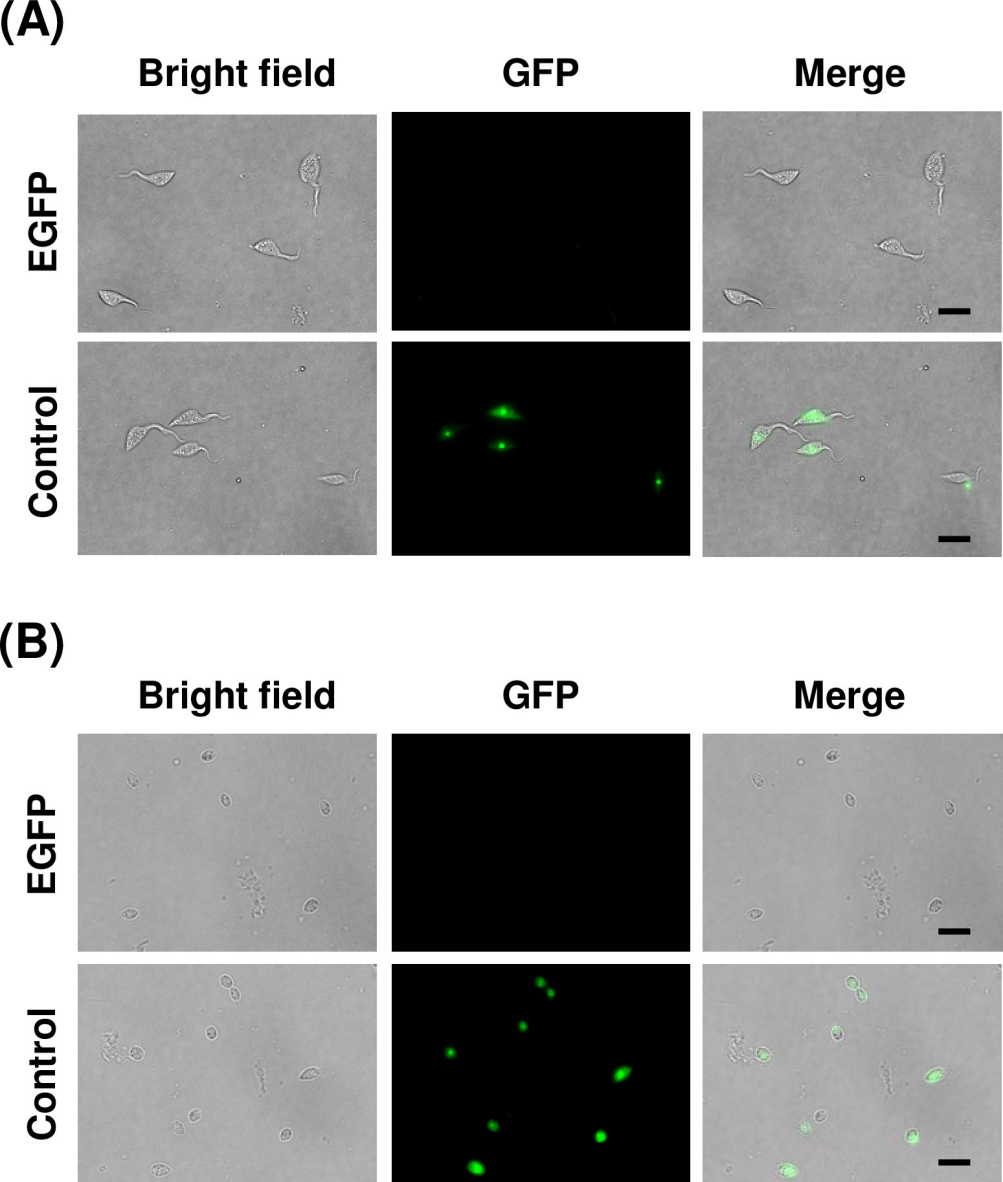
CRISPR/Cas9-mediated knockout of EGFP. (A) gRNAs against *EGFP* and control gRNA were transfected into epimastigote of Cas9 cell line. The pictures were taken at 1 day after electroporation. Scale bars: 10 μm. (B) gRNAs against *EGFP* and control gRNA were transfected into EA derived from Cas9-ama cell line. The pictures were taken at 1 day after electroporation. Scale bars: 10 μm.

To this end, we investigated whether CRISPR/Cas9 system can function in EA to knockout a target gene and yield measurable growth phenotype to allow evaluation of the target essentiality, either as an axenic culture or as intracellular amastigotes followed by host infection. For a proof of concept, we first transfected gRNA against *EGFP* into the stable Cas9 cell line, which harbors EGFP as a Cas9 fusion protein, to confirm the functionality of the system and to estimate the knockout efficiency (Fig 5). The fraction of EGFP-positive population dropped to 2% in epimastigote and to 4% in amastigote at 1 day post transfection. We routinely achieved knockout efficiency higher than 95% in both epimastigote and amastigote using conventional electroporation method, based on the fluorescence intensity. Analysis of genomic DNA confirmed that CRISPR/Cas9-mediated knockout introduced mutations in the target DNA. (S3 Fig).

**Fig 6:**
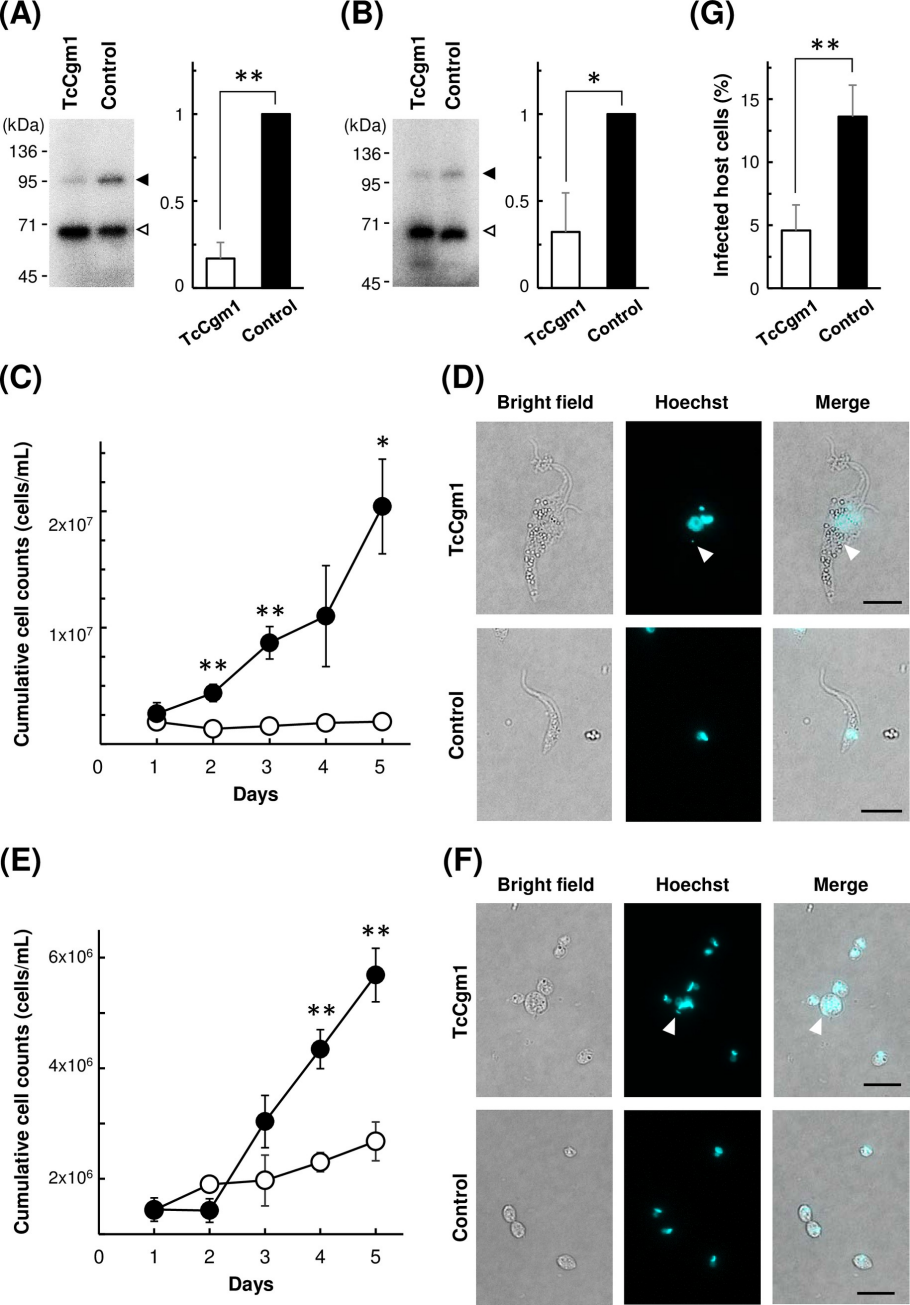
Phenotype of *TcCGM1*-knockout in epimastigote and amastigote. (A) Guanylyltransferase assay of *TcCGM1*-KO epimastigote and control epimastigote cell lysates. Upper band corresponds to radiolabeled TcCgm1 (118 kDa, black arrow head) and the lower corresponds to TcCe1 (67 kDa, white arrow head). Phorphorimage of SDS-polyacrylamide gel is shown. Positions of co-electrophoresed molecular weight marker is shown on the left. The amount of [^32^P]-labeled target protein relative to the control level is quantitated, and the average of four guanylyltransferase assays (±SD) from two independent knockout experiments are plotted on the right (**p<0.01, paired t-test). (B) Guanylyltransferase assay of *TcCGM1*-KO amastigote and control amastigote cell lysates. The amount of [^32^P]-labeled target protein relative to the control level is quantitated, and the average of four guanylyltransferase assays from two independent knockout experiments are plotted on the right (*p<0.05, paired t-test). (C) Growth curve of Cas9-expressing epimastigote after transfection with *TcCGM1*-gRNA (open circle) and control gRNA (closed circle). Electroporations were performed in triplicates, and mean values (±SD) are plotted. Significant difference compared to control is indicated (**p<0.01, *p<0.05, Welch’s t-test). (D) Morphology of epimastigotes after transfection with *TcCGM1*-gRNA (upper panel) and control gRNA (lower panel). Images were taken at 3 days post transfection. Scale bars: 10 μm. (E) Growth curve of Cas9-expressing axenic amastigote after transfection with *TcCGM1*-gRNA (open circle) and control gRNA (closed circle). Electroporations were performed in triplicates, and mean values (±SD) are plotted. Significant difference compared to control was indicated (**p<0.01, Welch’s t-test). (F) Morphology of axenic amastigotes after transfection with *TcCGM*1-gRNA (upper panel) and control gRNA (lower panel). Images were taken at 5 days post transfection. Scale bars: 10 μm. (G) Infection efficiency of *TcCGM1*-KO amastigotes (white bar) and control amastigotes (black bar) to host 3T3 cells. The average of three infection experiments (±SD) are plotted (**p<0.01, Welch’s t-test).

We then targeted an endogenous gene, *TcCGM1* as a model target. TcCgm1 is *T*. *cruzi* homologue of *T*. *brucei* mRNA capping enzyme TbCgm1, which is responsible for cap 0 formation on SL RNA and is essential for the proliferation of *T*. *brucei*[28]. We transfected gRNA against *TcCGM1* into epimastigote and amastigote stages of *T*. *cruzi*. Guanylyltransferase activity of TcCgm1, along with the other capping enzyme TcCe1, can be detected in the cell lysate by incubating the total protein with [α-^32^P]-GTP in presence of metal cofactor. The resulting enzyme-[^32^P]-GMP covalent intermediate can be visualized as a radiolabeled band in SDS-polyacrylamide gel. After transfection with gRNA against *TcCGM1*, signal of TcCgm1-[^32^P]-GMP became weak comparing to the control cells that received gRNA with unrelated sequence (Fig 6A and 6B). Radiolabeled signal of the other guanylyltransferase, TcCe1, was relatively unaffected. The amount of TcCgm1-[^32^P]-GMP was reduced to 32% in epimastigote and to 49% in axenic amastigote 2 days after gRNA transfection.

We then monitored the phenotype of *TcCGM1* knockout cells after gRNA transfection. In epimastigote, Cas9 cells transfected with *TcCGM1*-gRNA halted the growth on the day after electroporation (Fig 6C). Deformation of the knockout cells started to appear on day 2, and became clearly noticeable on day 3 post transfection (Fig 6D). *TcCGM1*-knockout epimastigotes tended to be large, and often possessed multiple flagella. Nuclear staining revealed that many cells contained abnormal number or size of nuclei or kinetoplasts. Much smaller spots of unknown nature were observed in some cases (Fig 6D, arrow head). In axenic amastigote, growth of Cas9-ama cells transfected with *TcCGM1*-gRNA was also suppressed (Fig 6E). For the first 3 days, there was no apparent morphological changes in knockout cells, comparing to the amastigotes received control gRNA. However from day 4, *TcCGM1*-knockout amastigotes started to display irregular shapes. Deformation became more noticeable on day 5 (Fig 6F). Unlike knockout epimastigotes, not many cells possessed multiple nuclei or kinetoplasts, except occasional large cells that show abnormal Hoechst staining pattern (Fig 6F, arrow head).

For intracellular amastigote assay, gRNA-transfected EAs were applied onto 3T3 cells 1 day after electroporation at MOI of 20, and allowed to infect host cells for 2 days. Amastigotes remained outside of the host cells were washed away, and host-parasite co-culture was incubated for additional 2 days. Infected 3T3 host cells were then fixed and stained by Hoechst to identify intracellular amastigotes (S4 Fig) to calculate the percent infection. Fraction of host cells infected by amastigotes transfected with *TcCGM1*-gRNA and control gRNA were 4.6% and 13.6%, respectively (Fig 6G). This outcome is in agreement with the transfectants’ cell growth monitored as axenic cultures (Fig 6E). Taken together, these results show that essentiality of a target gene in EA can be analyzed after CRISPR/Cas9-mediated knockout by monitoring growth phenotype of the amastigotes, either as axenic culture or as intracellular amastigotes followed by host invasion.

## Discussion

*T*. *cruzi* goes through distinct life cycle stages as they travel between insect vectors and mammalian hosts. Being able to isolate and study individual stage is crucial in understanding the parasite biology. Epimastigote stage of *T*. *cruzi* has been routinely used for basic cell biology research and drug development study, because of the easiness of culture maintenance. On the other hand, amastigote had received little attention as a subject of direct experimental manipulation due to complication and inaccessibility in the host co-culture, even though this life cycle stage is most relevant in host-parasite interaction and drug development studies.

Here, we demonstrated that EA can be proliferated as axenic culture at least for one week without major morphological change or loss of stage-specific gene expression. Susceptibility of EA and intracellular amastigote to benznidazole and nifurtimox was comparable in terms of EC50 values. We also demonstrated that a plasmid vector can be delivered directly into EA for transient gene expression, and transfectants can infect host 3T3 cells and replicate just like *bona fide* amastigotes even after 6 days of axenic culturing in presence of selective agent. CRISPR/Cas9 system can function in Cas9-expressing axenic amastigotes when gRNA is transfected by conventional electroporation. These new methodologies open up the possibility to carry out stage-specific experiments in a truly amastigote-specific manner.

It is widely accepted that amastigote of *T*. *cruzi* is an obligate intracellular parasite. Host metabolic factors such as Coenzyme Q_10_ and Akt-related pathways including glucose and lipid metabolisms have been implicated as key growth regulators of intracellular amastigote[45,46]. Our result indicates that components in LIT medium can compensate for such nutrient needs, at least for a short while, to sustain the growth of axenic amastigote. Although it was previously demonstrated that EA can uptake exogenous glucose[47], it is unlikely that glucose by itself allows axenic proliferation, because DMEM contains higher concentration of glucose than LIT medium, yet EA kept in DMEM did not replicate at all (Fig 1A). Optimum nutrients required for axenic amastigote cultivation beyond 1 week remain to be investigated. Also, whether the technique of genetic manipulation during temporal axenic cultivation is applicable to other strains of *T*. *cruzi* or not must be investigated in future studies. There are few instances in previous literatures that EA increased in number during host-free incubation in Y strain[30] and Brazil strain[10,29], but those observations were not followed up.

Recently, several techniques for high-throughput inhibitor screening against *T*. *cruzi* host co-culture have been developed (Reviewed in [37]). However, those systems are specialized for phenotypic assays in compound screenings. In order to identify a drug target, to probe into a mode of action of drug candidates, or to investigate the biological role of specific gene, there is still a great need to directly investigate the amastigote itself. Preceding examples of utilization of axenic amastigotes can be found in *Leishmania* drug screening studies[48–53]. It must be noted, however, that inhibitory compounds identified by axenic assay and host co-culture assay do not perfectly overlap[49,52]. This discrepancy originates in part from physiological differences between intracellular and extracellular forms of *Leishmania* amastigotes, namely proteome[54] and transcriptome[55]. In *T*. *cruzi*, some differences between the two forms of amastigotes have also been reported. For example, EA is more resistant to complement-mediated lysis than intracellular amastigote. Hundred percent of intracellular amastigote is lysed in fresh serum but EA is completely resistant in case of Tulahuen strain[56]. Also, EA is more infectious to the cultured host cell than intracellularly-derived amastigotes[56]. Since literature on drug treatment of *T*. *cruzi* axenic amastigotes is extremely limited[29] and this present study is the first instance of directly comparing the dose response curves of EA to that of intracellular amastigotes in host cells, it definitely requires further investigations to see whether *T*. *cruzi* axenic amastigotes can be utilized for inhibitor screening assays in general. Our data indicate that Hill Slopes tend to be shallower in axenic amastigote comparing to intracellular amastigote (Fig 3). This might partly be resulted from different counting schemes in our experiments, i.e., resazurin assay of axenic amastigote reflects redox activity of viable parasites, whereas percent host infection of intracellular amastigote does not account for the population dynamics of the parasite within individual host cell. Alternatively, steepness of the dose response curves may be associated with complexity of the target molecule or pathway, and presence or absence of the host cells could affect “tipping point” of the lethality. Since benznidazole and nifurtimox both affect wide range of cellular machinery by generation of reactive oxidant species, it would be interesting to see whether target-specific trypanocidal compounds also produce similar slope trends in dose response profiles. If significant phenotypic discrepancies are found between EA and intracellular amastigotes in drug sensitivity or selectivity, those inhibitors potentially provide valuable insights into host-parasite interaction and cellular biology of *T*. *cruzi* amastigote. Considering the amount of information we can extract, there is no doubt that parasite-host co-culturing is the most relevant system in terms of phenotypic assay[37]. It surely requires further investigation to determine the relevance and practicality of the use of EA in drug screening. Nonetheless, the use of EA can give us an easy and fast evaluation of compound susceptibility of amasitigote itself. In the co-culture system, passing the host cell barrier is one of the top criteria that the compounds are selected for. However, it is not uncommon that subsequent chemical modification significantly improves the permeability of the compound to cell membrane through lead optimization process[57–59] or by drug delivery system[60]. Therefore, the use of naked amastigote in early-stage compound screening may give chance to some candidate compounds that are otherwise dropped out, and expand the options of starting materials to move forward with the next step of drug development.

There are some studies utilizing EA in *T*. *cruzi* in the past, however their objectives were mostly limited to investigation of signaling factors involved in trypomastigote-to-amastigote differentiation[30,61] or host invasion[16–20]. To our knowledge, the present study is the first instance of utilizing *T*. *cruzi* EA for direct transfection for exogenous gene expression and endogenous gene knockout. Previously, Padmanabhan *et al*. demonstrated that trypomastigote can be transfected for later infection and differentiation to produce transgene-expressing intracellular amastigotes[6]. In their report and also in our hands, plasmid transfection efficiency of trypomastigote is about 5%. One advantage of using EA instead of trypomastigote is that electroporation can be followed by proliferation and selection of transfectants to enrich positive population to compensate for low transfection efficiency. In our present study, fraction of mDsRed-positive EA was initially about 4%, but reached to 37% after 5 days of blasticidin selection (Fig 4B). It may be possible to improve the enrichment efficiency by using a selection marker that requires shorter selection period, or by improving the culture medium to allow longer cultivation of axenic amastigote. It is, of course, feasible to take advantage of a fluorescence activated cell sorter prior to the host infection[6] to obtain homogeneous population of transgenic amastigotes instead.

Another advantage of EA transfection is that we can bypass active host invasion (as supposed to “passive” mode of infection by amastigotes) and trypomastigote-to-amastigote differentiation that may introduce unwanted bias to transfectants, which is crucial when studying stage-specific cellular functions. For example, in a drug target research, one would like to produce knockout parasites in search for a target gene that is essential in clinically-relevant amastigote stage. However, if a candidate gene was essential in trypomastigote stage, we cannot obtain a knockout amastigote by infection and differentiation of the lethal trypomastigote. Our strategy of using axenic amastigotes enables evaluation of essential genes truly in amastigote-specific manner. Knockout study of some target genes may yield different outcomes between axenic amastigotes and host intracellular amastigotes. If so, those cases provide us with opportunities to examine the involvement of host factors in amastigote-specific gene functions.

In summary, having direct access to amastigote as experimental tools may greatly expand methodological freedom to investigate basic cellular biology of *T*. *cruzi* and potentially provide valuable insights into the drug development study in the future.

## Supporting information

S1 Fig Expression vectors used in this study. Schematic representations of plasmid constructs for expression of EGFP, Cas9-EGFP, mDsRed, and stage-specific expression of EGFP and Cas9-EGFP. Names of constructs are indicated on the top of each partial plasmid map. Names of cell lines produced by transfection of corresponding plasmid are indicated on the left. Neo, neomycin resistance gene; Bsd, blasticidin resistant gene; 3’UTR, amastin 3’UTR.
(TIF)

S2 Fig EGFP expression of EGFP and EGFP-ama cell lines in epimastigote, trypomastigote and intracellular amastigote stages. (A) Epimastigote (B) Trypomastigote (C) Intracellular amastigote.
(TIF)

S3 Fig Analysis of genomic DNA after CRISPR/Cas9-mediated *EGFP* knockout. (A) Detection of mutations with T7 endonuclease I. Genomic DNA was extracted from Cas9-expressing epimastigote (Epi) and amastigote (Ama) 2 days after transfection with control gRNA or EGFP-targeted gRNA. EGFP fragment was PCR-amplified, re-annealed, and digested with T7 endonuclease I to detect mutations in the target sequence. Position of cleaved PCR product is indicated by an arrow. (B) Sequence of deletion mutant. PCR product from Ama EGFP-knockout in (A) was ligated into a plasmid by TOPO TA cloning to analyze the sequence. Identified deletion is highlighted in yellow. Positions of crRNA complementary sequence and PAM sequence are indicated by blue and green boxes, respectively.
(TIF)

S4 Fig Infection of host 3T3 cells by *TcCGM1*-knockout amastigotes. (A) EA derived from Cas9-ama cell line was transfected with gRNA against *TcCGM1*, and was applied onto host 3T3 cells immediately after electroporation. Amastigotes remained outside of the host cells were washed away after 2 days. The culture was incubated for additional two days before formalin fixation and staining with Hoechst 33342. (B) EA derived from Cas9-ama was transfected with control gRNA, and transfectants were used to infect host 3T3 as described in (A).
(TIF)

S1 Table List of primers used for qPCR. Target gene name and ID, sequence and position of oligonucleotides are summarized.
(TIF)

S2 Table Transfection efficiency in axenic amastigotes of *T*. *cruzi* with various electroporation pulse programs. Mean values (±SD) of 3 independent experiments are shown.
(TIF)

S1 Movie Amastigote-to-trypomastigote differentiation of blasticidin-selected mDsRed-expressing parasite (Bright field). (AVI)

S2 Movie Amastigote-to-trypomastigote differentiation of blasticidin-selected mDsRed-expressing parasite (mDsRed). (AVI)
