# Supplementary figures and images for: Utilization of proliferable extracellular amastigotes for transient gene expression, drug sensitivity assay, and CRISPR/Cas9-mediated gene knockout in Trypanosoma cruzi
Source: PLoS Negl Trop Dis. 2019 Jan 14;13(1):e0007088. doi: 10.1371/journal.pntd.0007088 (PMC6347291; doi:10.1371/journal.pntd.0007088)

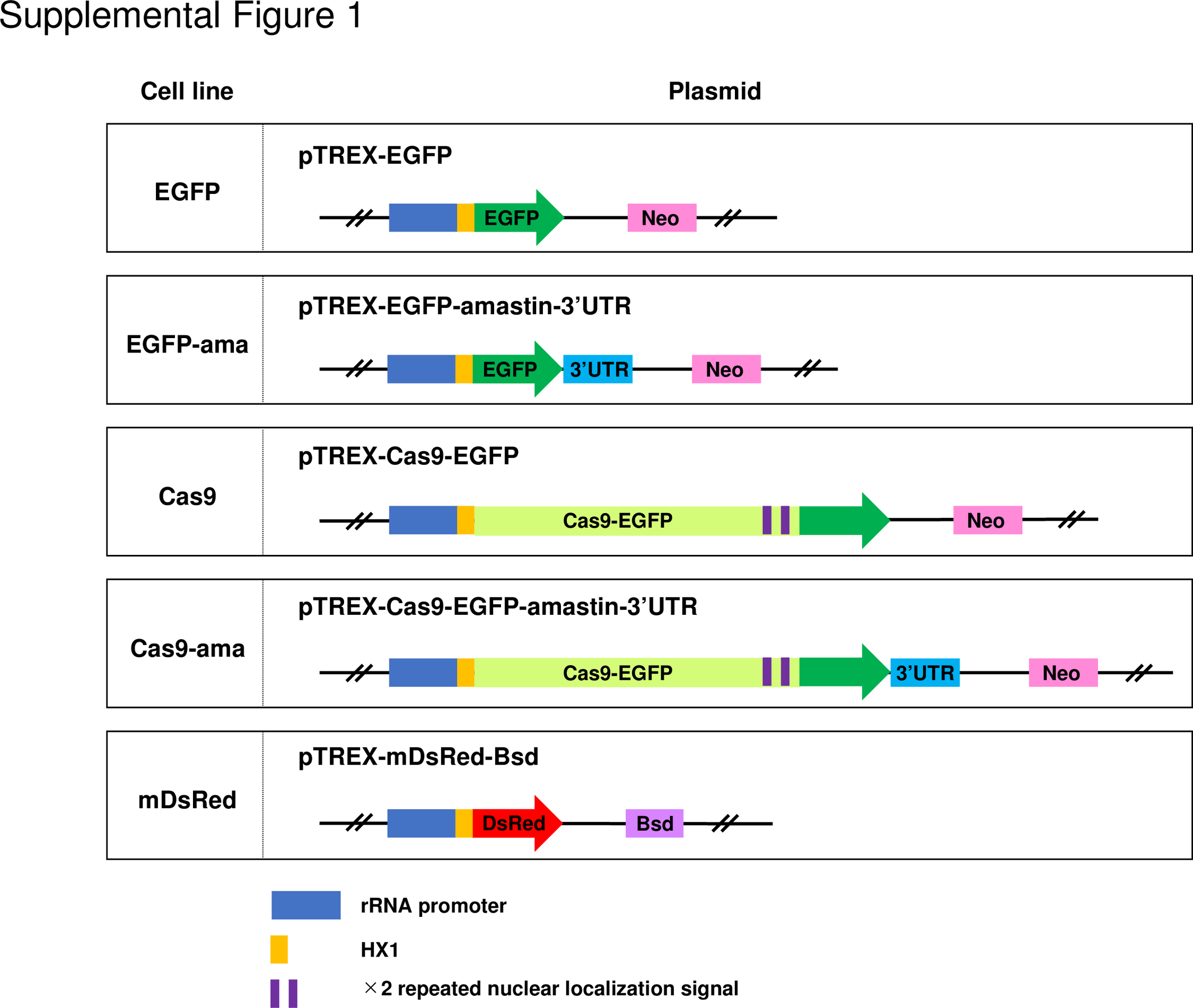

Supplement: S1 Fig — Schematic representations of plasmid constructs for expression of EGFP, Cas9-EGFP, mDsRed, and stage-specific expression of EGFP and Cas9-EGFP. Names of constructs are indicated on the top of each partial plasmid map. Names of cell lines produced by transfection of corresponding plasmid are indicated on the left. Neo, neomycin resistance gene; Bsd, blasticidin resistant gene; 3’UTR, amastin 3’UTR. (TIF) [file pntd.0007088.s001.tif]

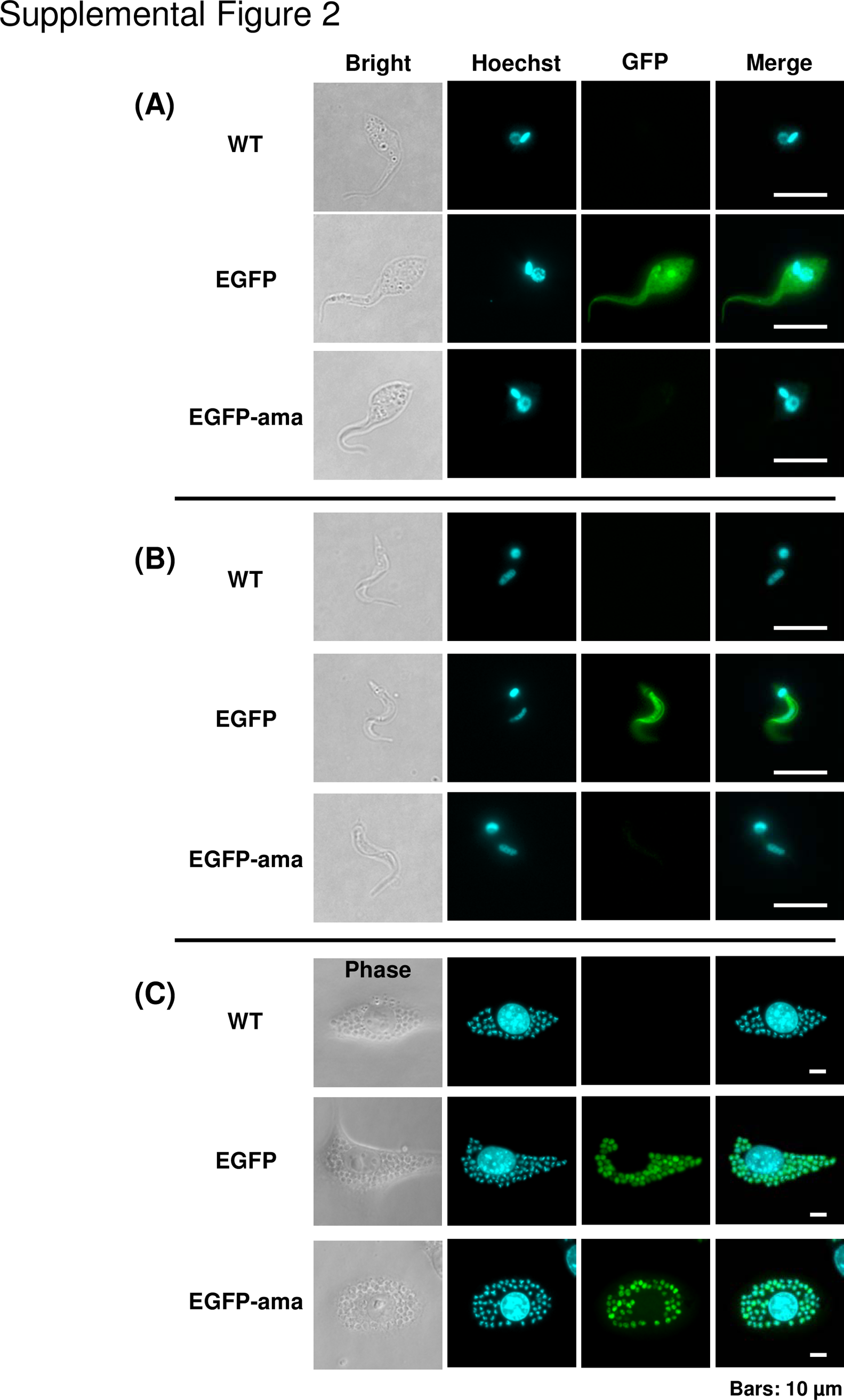

Supplement: S2 Fig — (A) Epimastigote (B) Trypomastigote (C) Intracellular amastigote. (TIF) [file pntd.0007088.s002.tif]

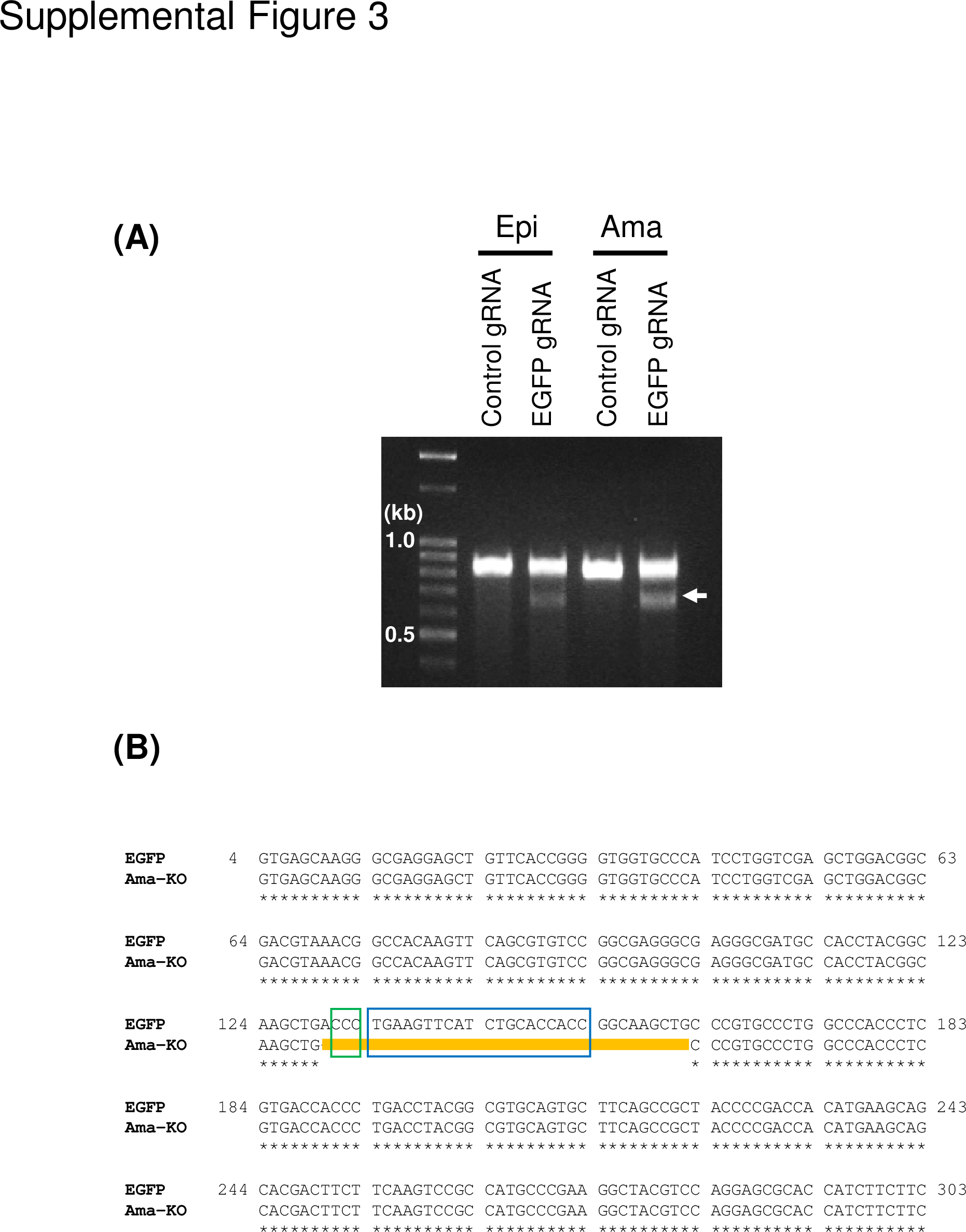

Supplement: S3 Fig — (A) Detection of mutations with T7 endonuclease I. Genomic DNA was extracted from Cas9-expressing epimastigote (Epi) and amastigote (Ama) 2 days after transfection with control gRNA or EGFP-targeted gRNA. EGFP fragment was PCR-amplified, re-annealed, and digested with T7 endonuclease I to detect mutations in the target sequence. Position of cleaved PCR product is indicated by an arrow. (B) Sequence of deletion mutant. PCR product from Ama EGFP-knockout in (A) was ligated into a plasmid by TOPO TA cloning to analyze the sequence. Identified deletion is highlighted in yellow. Positions of crRNA complementary sequence and PAM sequence are indicated by blue and green boxes, respectively. (TIF) [file pntd.0007088.s003.tif]

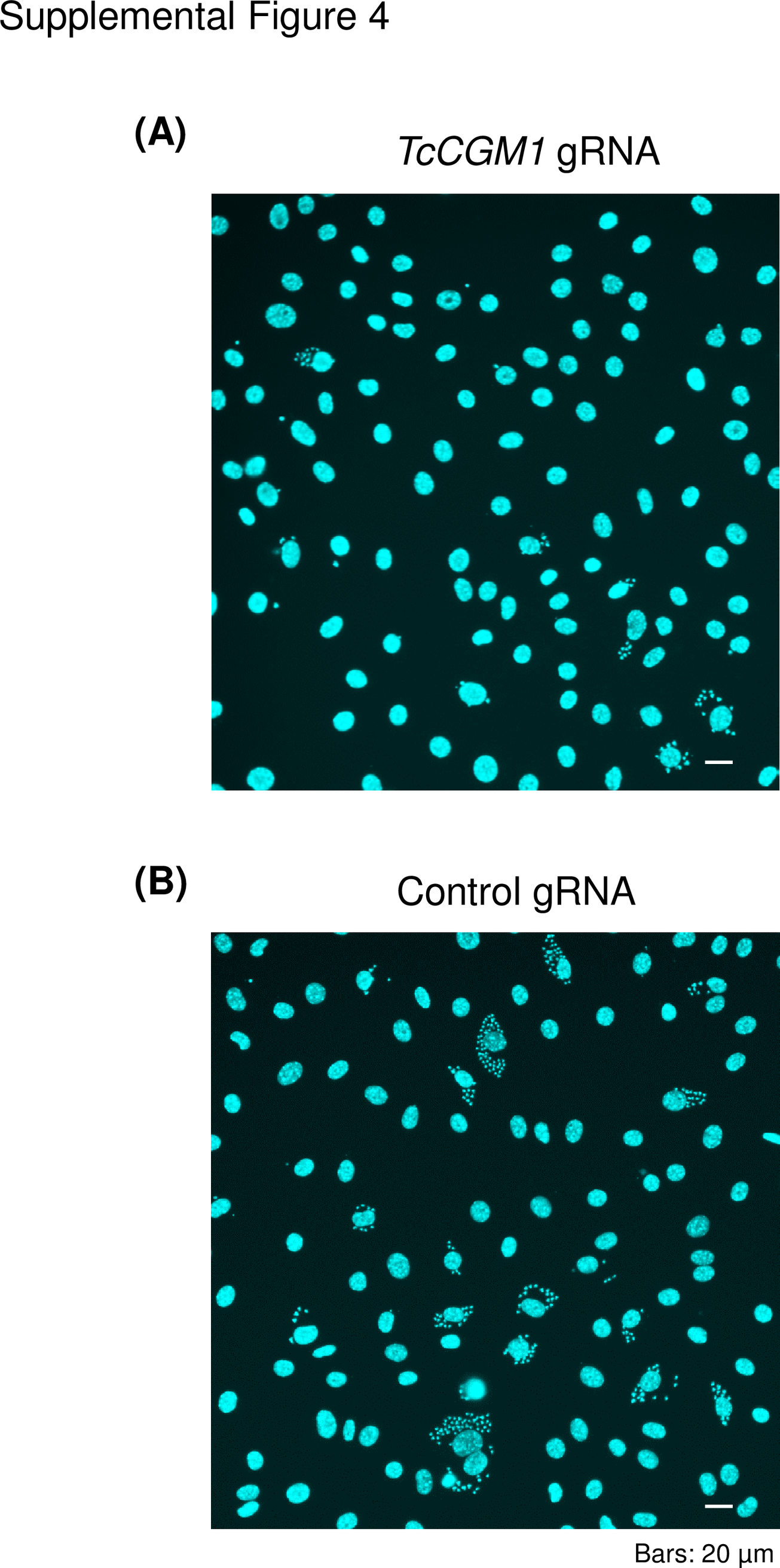

Supplement: S4 Fig — (A) EA derived from Cas9-ama cell line was transfected with gRNA against TcCGM1, and was applied onto host 3T3 cells immediately after electroporation. Amastigotes remained outside of the host cells were washed away after 2 days. The culture was incubated for additional two days before formalin fixation and staining with Hoechst 33342. (B) EA derived from Cas9-ama was transfected with control gRNA, and transfectants were used to infect host 3T3 as described in (A). (TIF) [file pntd.0007088.s004.tif]

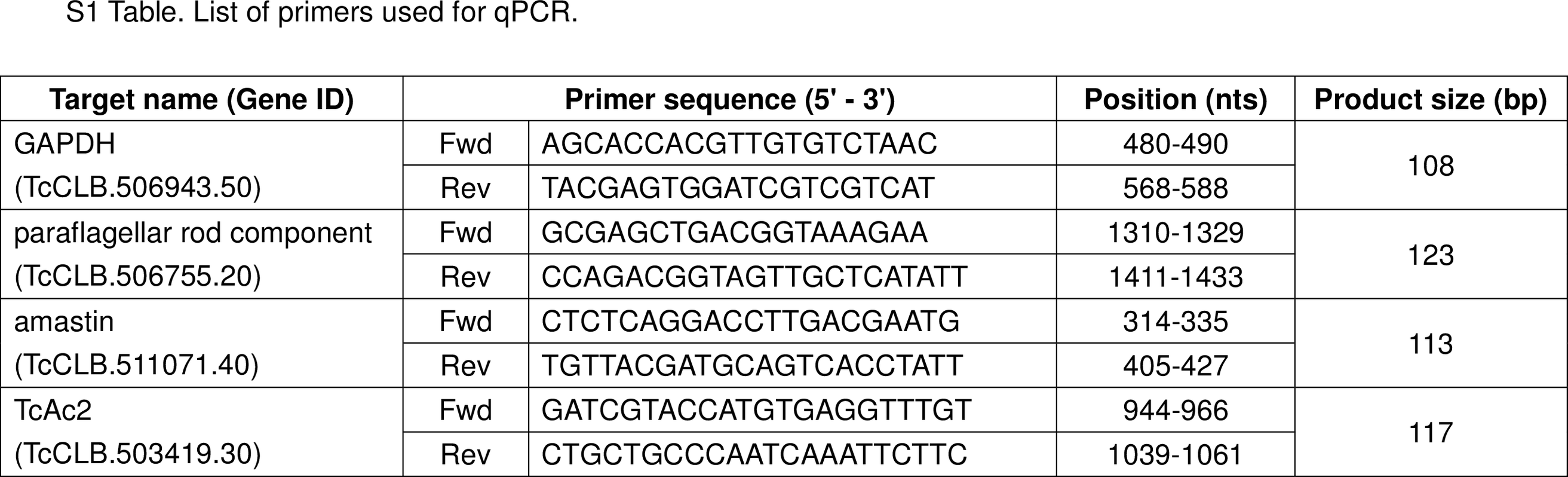

Supplement: S1 Table — Target gene name and ID, sequence and position of oligonucleotides are summarized. (TIF) [file pntd.0007088.s005.tif]

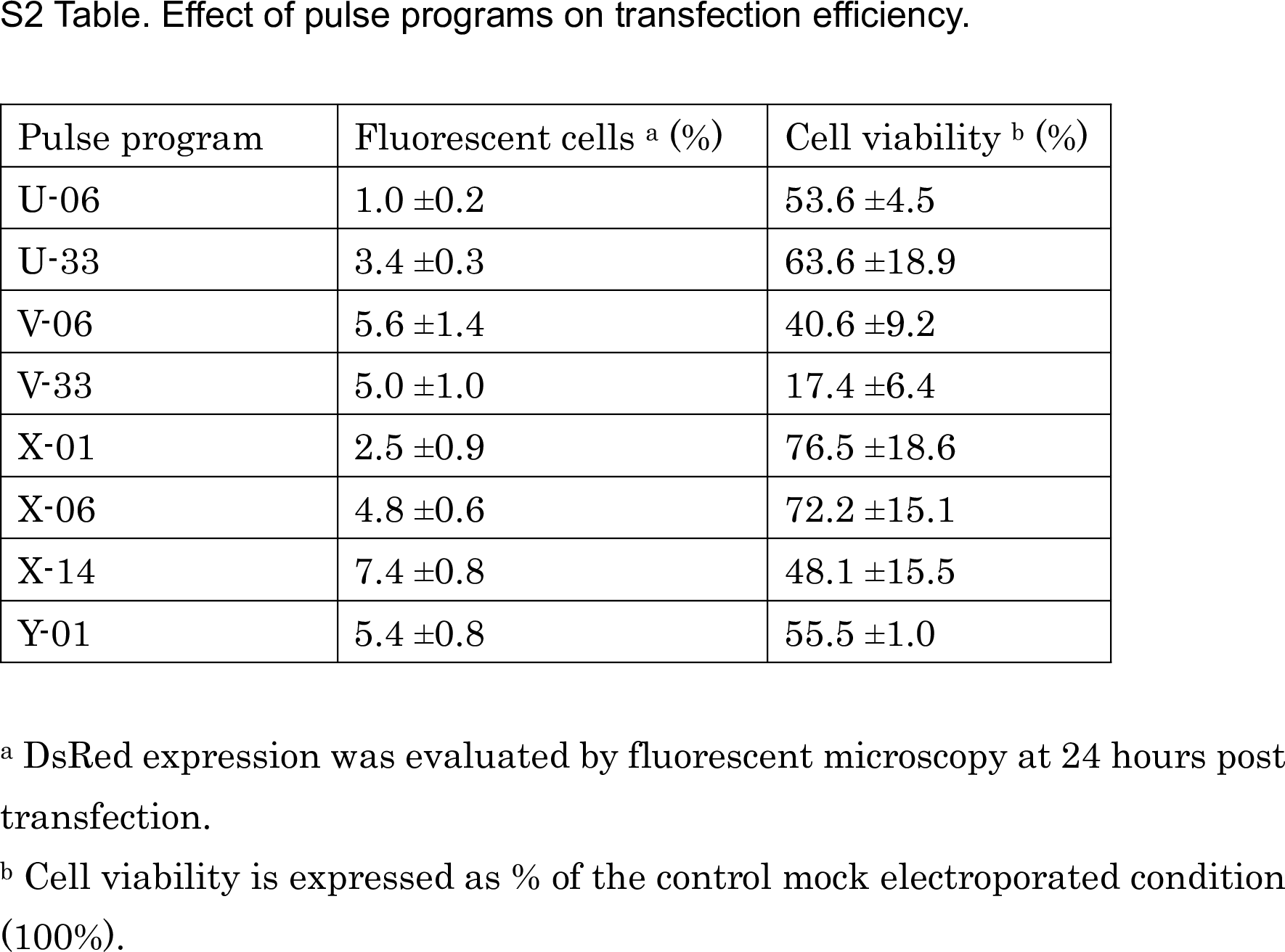

Supplement: S2 Table — Mean values (±SD) of 3 independent experiments are shown. (TIF) [file pntd.0007088.s006.tif]
